# The Quantity Index × Quality Index (Q×Q) Model for Ornamental Value Assessment in Garden Roses Based on Rose Hips, and the Evaluation of 704 Field-Grown Cultivars and Taxa

**DOI:** 10.3390/plants15162515

**Published:** 2026-08-20

**Authors:** Gábor Boronkay, András Neményi, Szilvia Kisvarga, Katalin Horotán, László Orlóci

**Affiliations:** 1 Institute of Landscape Architecture, Urban Planning and Garden Art, Hungarian University of Agriculture and Life Sciences (MATE), Páter Károly út 1., H-2100 Gödöllő, Hungary; nemenyi.andras.bela@uni-mate.hu (A.N.); kisvarga.szilvia@uni-mate.hu (S.K.); orloci.laszlo@uni-mate.hu (L.O.); 2 Zoological Department, Institute of Biology, Eszterházy Károly Catholic University, H-3300 Eger, Hungary; horotan.katalin@uni-eszterhazy.hu

**Keywords:** CIEL*a*b*, colorimetry, decorativeness, pseudo-fruit, rose-hip

## Abstract

A model was developed to calculate the rose-hip-based ornamental value (OV_hip_) of rose cultivars. In this “Quantity Index × Quality Index” (Q×Q) model, the Quantity Index refers to hip production, whereas the Quality Index refers to the epicarp color of the rose hip. The rapid determination of quantity under field conditions requires ranking, which provides only an approximate estimate; however, its precision can be improved. To replace ranking class numbers with production-based values, a sample of items was both ranked (C_hip_) and measured (P_hip_). A strong power–function relationship (P_hip_ = 0.12792 × C_hip_^3.00120^) was found; therefore, the calculated P_hip_ values can be used as the Quantity Index. To determine the Quality Index, the aesthetic value of 21 rose hip samples was scored, and the epicarp colors of the same samples were also measured. A strong linear correlation was found between the scores and the a* coordinate of the CIE color space, consequently; the CIE a* can be used as the Quality Index. In this Q×Q model, OV_hip_ is calculated as the product of these two indices after normalization. Using this model, 704 items were evaluated in the Budatétény Rose Garden (Hungary), where the highest OV_hip_ values were recorded for *Rosa micrantha*, *Rosa canina* ‘Inermis’, and the lucieae hybrid ‘Fragezeichen’ (Boettner, 1910). Among the hybrid tea roses, ‘DELamo’ (Delbard, 2004) and, among the floribundas, ‘Méphisto’ (Mallerin, 1951), had the highest OV_hip_.

## 1. Introduction

Among temperate ornamental shrubs, the cultivated rose (*Rosa × hybrida* or *Rosa* hybrid cultivars [1]), a complex interspecific hybrid, is probably the most diverse [2], as it is the result of several thousand years of cultivation and breeding. The earliest record of its use dates back to the 23rd century BCE, to the reign of Sargon I, as indicated by the Assyrian legend of Šar Tamḫāri, according to which the ruler brought roses from Anatolia to his Mesopotamian homeland for trial [3].

The antiquity of the species group is well characterized by the fact that it is based on more than 8 basic species [4] (the number is 9 or 10 depending on taxonomic revisions), and over the years, at least 20 more species have been included in its breeding, which is why the number of varieties is incredibly high. The number of rose cultivars officially registered by the American Rose Society (ARS), which serves as the International Cultivar Registration Authority for Roses, has now exceeded 37,000 [5]. However, if amateur breeding is also taken into account, the total number of cultivars ever created may be as high as 45,000 or more. For example, Kmecl estimates the number to be between 50,000 and 60,000 [6]. At the same time, not only the number of cultivars but also their phenotypic variability is unparalleled. The cultivars are complex hybrids of multiple alloploid species [7], but even the monophyleticity of these basic wild species is questionable [8,9].

Despite this unprecedented complexity, horticulturists must be able to compare varieties, which is not an easy task, since—unlike food crops—ornamental plants are usually evaluated primarily by characteristics that are difficult to quantify and often difficult to define as well. Such traits include the planting value, ornamental value, or climate tolerance, all of which should be evaluated for each cultivar according to a uniform set of criteria, even though the differences between cultivars may be extraordinarily large. A good example is that the micro-miniature cultivar ‘Si’ reaches a maximum height of about 25 cm, whereas in the case of ‘Paul’s Himalayan Musk Rambler’, this value exceeds 900 cm [5]. In the case of this bewilderingly complex hybrid group, however, the evaluation of cultivars must be solved, since roses are relatively short-lived yet expensive ornamental shrubs, which, through their flowering, foliage, and fruit, always constitute a prominent element of gardens and parks. Therefore, selecting the most valuable cultivars and finding harmony between cultivars under given climatic conditions is a matter of considerable economic importance.

The Budatétény Rose Garden (Budapest, Hungary) has been working for decades to evaluate, quantify, and typify this decorative potential. Due to its favorable position, it is ideally suited for objective cultivar evaluation work, owing to its large number of cultivars, its mixed planting material (historical, modern, and lesser-known Hungarian cultivars), and its organizational structure. In line with one of the institution’s main research directions, we are working to make the decorativeness of rose cultivars measurable, although it is traditionally being regarded as an extremely subjective feature. We intend to evaluate the cultivation value of the varieties using complex indices, in order to ensure that the results are as objective as possible.

This requires data types that allow even very different varieties to be compared. However, at the same time, it is also a requirement that even large cultivar collections have to be evaluated in the field within a relatively short period of time. This time window may generally be no more than 10 days, while the number of items to be assessed may reach several thousand. The short data collection period is necessitated by the variability of the weather and the plant ontogeny. The task here, therefore, is not to develop evaluation methods for artificial climates under controlled laboratory conditions, but rather to elaborate on procedures that can be applied in outdoor plantations, test gardens, and cultivar collections. It is essential that these evaluations provide exact results based on mathematical statistics; however, only rapid, large-scale open-field data collection is possible.

### 1.1. General Q×Q Model

In order to make it possible to evaluate a cultivar collection, we developed an ornamental value calculation system, which, in the generalized form, was named the “Quantity Index × Quality Index” (or “Q×Q”) model. This two-index system, with slight adaptation, is suitable for calculating various decorative characteristics produced by different plant parts, as it is based on a similar logic. The model is founded on the principle that the ornamental value based on a plant organ (flower, leaf, fruit, thorn, bark, etc.) can be evaluated objectively if it is divided into two independent and quantifiable factors: the quality of a piece of plant organ responsible for decorativeness and the quantity (production) of this organ. The first characteristic forms the basis of the Quality Index, while the second forms the Quantity Index.

According to our hypothesis, the Quality Index can be derived from colorimetric data, as the color measurement is fast, precise, objective, suitable for calculations, and also aesthetically appropriate. The main question here is which colorimetric parameter or parameters describe the decorativeness most accurately. In some cases, this is evident, but where it is not, this can be determined by examining the correlation between personal judgments of aesthetic value and the measured colorimetric values. The color parameter that shows the strongest correlation can be selected. However, the Quantity Index cannot be measured with such precision. Due to the short data-collection time window, this value can only be ranked (classified), and it can only be regarded as an estimate, and therefore, it is not fully objective. However, the objectivity of the ranking can be improved: the definitions of these classes must be based on well-quantifiable values, and the number of classes must be as high as the conditions of field data collection allow. According to our hypothesis, data distortion can also be reduced if the actual, measurable quantitative production characteristic of each class can be calculated. To achieve this, the relationship between the class numbering used in the assessment and the actual calculated production values must be determined through regression analysis based on a small but representative sample. With the help of the regression formula, the class numbering can be replaced by the production value calculated from that class numbering. This approach particularly improves the accuracy of data collection because—as our preliminary experience shows—the relationship is usually not linear but follows a power or exponential function.

The Q×Q model does not provide information about the overall condition of the plant; rather, it attempts to quantify the ornamental value of specific plant parts that determine certain aesthetic value (blooming capacity, green space creation ability, etc.). Therefore, the model must be adapted to evaluate the decorativeness provided by different plant organs. In the following, we present the adaptations of the Q×Q model to decorativeness based on rose hips.

### 1.2. Adaptation of the Q×Q Model to Hip-Based Decorativeness

Although the terminology “fruit-based decorativeness” [10] is also used in the case of roses, the expression is not entirely accurate, since the rose hip is a pseudo-fruit closed at the top, in which the aggregate of achenes is enclosed by the fleshy hypanthium [11,12]. Although in many hybrid tea varieties, the pseudo-fruit is open and the achenes protrude, this is a deformity resulting from insufficient development of the hypanthium. Although the true fruit is not the rose hip but the achene, the aesthetic value of the varieties is nevertheless provided by the epicarp of the pseudo-fruit. Therefore, in our work, the model was designated as fruit-based or hip-based ornamental value (as an index: OV_hip_), since the term pseudo-fruit-based ornamental value (OV_pseudo-fruit_) seemed somewhat linguistically awkward.

#### 1.2.1. Quantity Index

**Figure 1:**
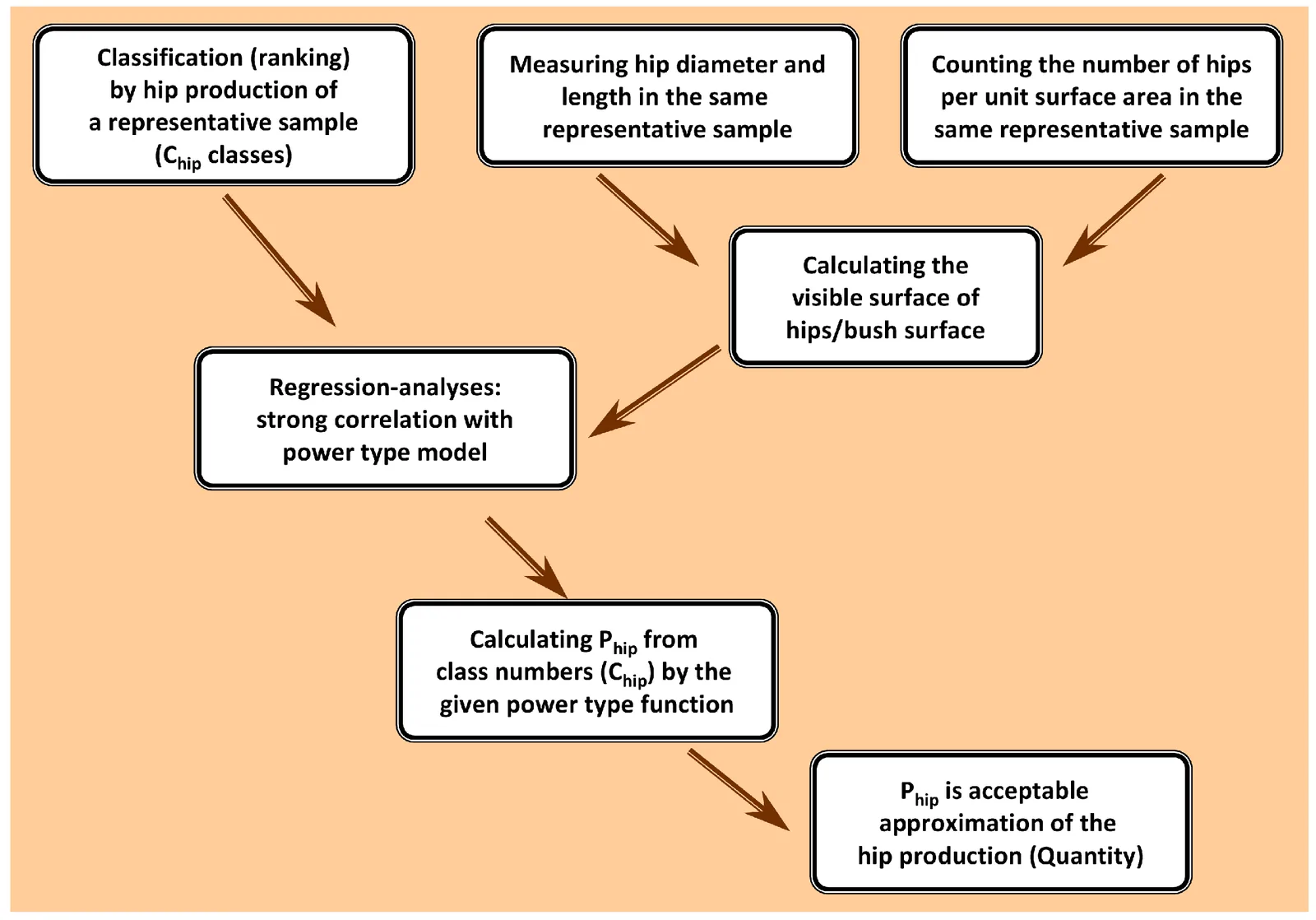
Flowchart illustrating the development of the Quantity Index of the Q×Q model. The figure illustrates the procedure used to calculate rose hip production and compares the calculated values with the corresponding estimated production. The flowchart presents the main steps of the procedure, while the details are provided in Section 1.2.3.

This index measures the production of the plant part that determines the decorativeness (Figure 1). In the case of hip-based decorativeness in roses, this can be expressed by the “visible total surface area of rose hips per bush surface area” (hip production: P_hip_). Due to the short time window forced by ontogenesis and changing weather conditions, the method of data collection is limited to classification, and only the serial numbers of classes (C_hip_ = 0, 1, 2, etc.) are available for further calculations. However replacing C_hip_ numbers with P_hip_ production values can be solved, as P_hip_ can be calculated based on the visible surface area of the individual rose hips, their quantity, and the degree of overlap. The visible surface area of a rose hip itself can be well approximated by its planar projection, which is the area of an ellipse here. However, this cannot be implemented when dealing with a large number of evaluations; instead, it must be calculated based on a small, representative sample. To reveal the systematic relationship between the estimated and ranked production (C_hip_) and the calculated production (P_hip_), non-linear regression analysis is required, since the relationship may follow a power or exponential pattern. If an appropriate relationship is found, its function will be suitable for replacing C_hip_ with P_hip_ for all classes. Having the function, the substitution can automatically be performed in subsequent data collection.

#### 1.2.2. Quality Index

**Figure 2:**
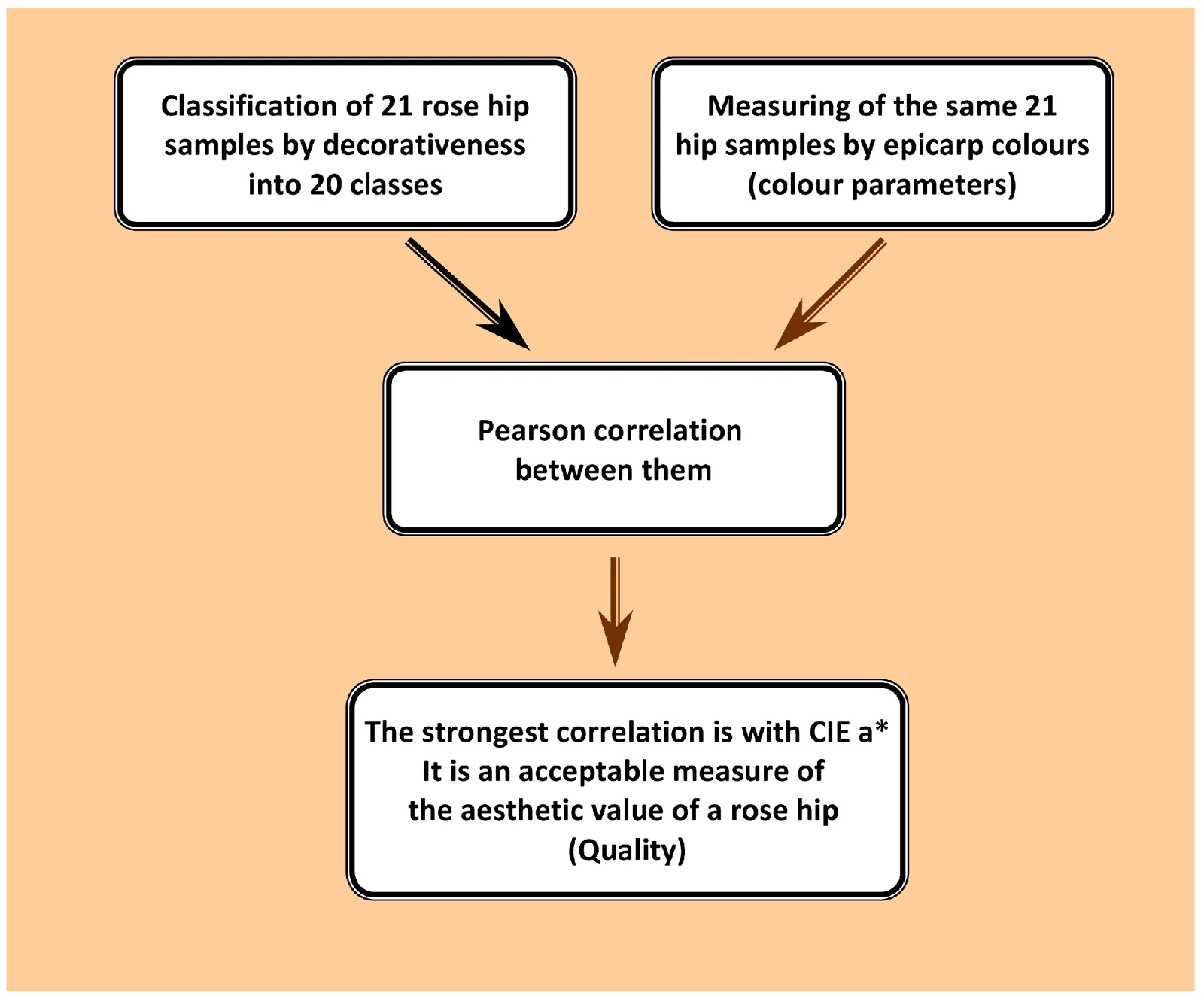
Flowchart illustrating the development of the Quality Index of the Q×Q model. The figure illustrates the procedure used to choose the most suitable variable for estimating the decorativeness of the rose hip epicarp. The flowchart presents the main steps of the procedure, while the details are provided in Section 1.2.3.

In the Q×Q model, the aesthetic value of a decorative plant part can be determined based on the color of the organ, which, in this case, is the epicarp of the rose hip (Figure 2). It can essentially be described by four parameters: the epicarp color of the rose hip, the epicarp texture, the shape of the pseudo-fruit, and the amount of dried sepals remaining on it. However, considering that the shape of the rose hip and the glossiness of its skin show relatively low variability and that the sepals visually blend into the appearance of the shrub, the epicarp color of the pseudo-fruit seemed sufficient to describe the ornamental value of a piece of rose hip. However, we also intended to verify this. This assumption is particularly important given that color can be measured quickly, precisely, and objectively, whereas fruit shape and glossiness are difficult to quantify, and the condition of the sepals can only be categorized.

Since, in roses, it is not evident which measured color parameter represents the decorativeness most accurately, the rose hip samples must be judged by independent evaluators in order to identify the strongest relationship between the judged aesthetic value and the measured colorimetric parameters. For this purpose, linear correlation analysis appears to be an appropriate method, assuming that among the many measurable color dimensions, there exists a parameter that shows strong correlation with the assessed decorativeness. The search for a nonlinear relationship is rendered almost unnecessary by the fact that the CIE color systems themselves already exhibit a certain degree of nonlinearity that is difficult to handle.

#### 1.2.3. Schematic Overview of the Development of the Q×Q Model Adapted to the Ornamental Value of Fruiting Plants

(A) Development of the Quantity Index (Figure 1)

(1) In a representative group of rose items, the samples are classified by estimated rose hip production. The resulting value is the sequence number of the class to which the item is assigned: C_hip_.
(2) For the samples of step (1), the spatial dimensions of the rose hips are measured, and from these, the visible surface area of the rose hips can be calculated, modeled as the area of an ellipse: A_hip_ = a_hip_ × b_hip_ × π.
(3) For the items of step (1), the number of rose hips per unit area is counted: N_hip._
(4) Based on steps (2) and (3), the ratio of the visible surface area of rose hips to the shrub surface area is calculated as the production value: P_hip_ = A_hip_ × N_hip_/A_bush_ × 100 (the unit is %_A/A_).
(5) Regression analysis is performed between the datasets X = C_hip_ and Y = P_hip_, and if the fit and reliability levels are adequate, the corresponding calculated P_hip_ value can be used instead of the class numbering (C_hip_).
(6) Normalization of P_hip_; this will serve as the Quantity Index.

(B) Development of a Quality Index (Figure 2)

**Figure 3:**
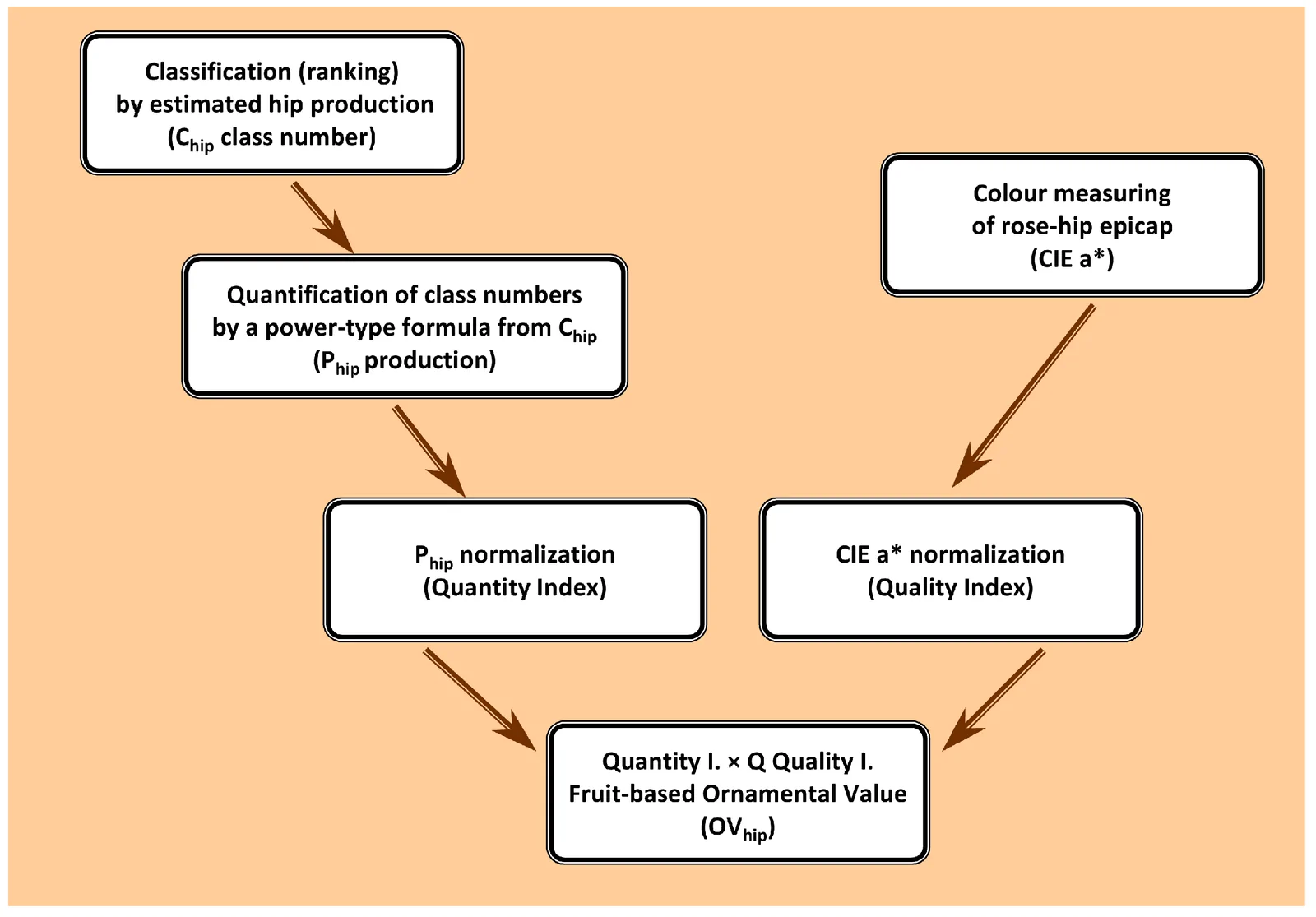
Flowchart illustrating the use of the Q×Q model to calculate the hip-based ornamental value of rose items. The figure illustrates the workflow for applying the Q×Q Ornamental Value model based on the Quantity and Quality indices. The flowchart presents the main steps of the procedure, while the details are provided in Section 1.2.3.

(7) Under laboratory conditions, rose hip samples with characteristic colors are scored for aesthetic value by independent evaluators.
(8) Correlation and factor analysis is used to assess the consistency of the evaluators’ work, and in the case of outliers (misinterpretations), the given evaluator’s data are excluded.
(9) Instrumental measurements are performed on the rose hips evaluated in step (7), and all measurable parameters are recorded (CIE L*a*b*, CIEDE2000, and the instrument’s unprocessed reflectance values).
(10) Using correlation analysis, the measured color parameter that is closest to the scored aesthetic value is selected. If a sufficiently strong correlation is found and the reliability of the model is acceptable, this parameter serves as the measurable dimension of quality.
(11) Normalization of the value determined in step (10) gives the Quality Index.

(C) Calculation of OV_hip_ (ornamental value of fruiting plants) as Quantity Index × Quality Index multiplication. Thus, the Q×Q model is ready.
(D) Application of the Q×Q Model (Figure 3): based on the completed Q×Q model, full-scale data collection can be carried out (both quantitative estimation and color measurement), followed by the calculation of indices and further processing of the results (mean, maximum, year effect, year-to-year stability, etc.).

## 2. Literature

### 2.1. General Literature Review

The rose’s pseudo-fruit, the hip, is a well-known agricultural product and has been recognized since antiquity as both a medicinal and a food plant. Its nutritional properties are described in hundreds of publications, which primarily praise its antioxidant effects, though the seeds are also effective. According to Turkish studies, it is particularly rich in unsaturated fatty acids [13]. Although the literature on the nutritional values of rose hips is extremely extensive, most works only briefly mention that it belongs to a group of species primarily used as ornamental plants [14].

Considering that the rose’s pseudo-fruit is edible, the pomological traits that are significant as an ornamental plant are also important breeding aspects as a medicinal plant. It is fortunate, because as a food crop, its productivity values are already agro-economic factors; therefore, several publications address the study of these characteristics. For example, Ostroshenko et al. [15] examined the number of rose hips of *Rosa rugosa* Thunb. per square meter, as well as its fresh and dry mass, depending on soil type. On sandy soil, yields were found to be only half compared to the control. Although the study does not mention it, their results can also be applied in ornamental horticulture, since rose hip production is a factor that also determines ornamental value. Gerçekcioğlu et al., in addition to analyzing the content values of the pseudo-fruit [16], conducted studies on the number of rose hips per bush and the mass of pseudo-fruit in varieties of *Rosa canina* L. and *Rosa montana* Chaix. In addition to the above-mentioned studies, the morphological variability of rose hips has also been investigated by numerous researchers, for example Stamin et al. [17] or Mariş and Ciulca [18]. However, most of these studies focus on local wild rose species or their selected varieties, as these are primarily used for consumption due to their smaller size and significantly higher productivity [19]. According to Janakiram and Debner [20], the size of the pseudo-fruits of wild species ranges between 0.5 and 2.8 cm in length and 0.1 and 2.7 cm in diameter. A publication closely related to the present study is that of Garbez et al. [21], where the fruit yield was not measured but classified, and they used a scale of 0–10 categories.

From the perspective of the Q×Q evaluation system that we developed, the most useful studies are perhaps those in which external coloration of pseudo-fruits was also recorded, in addition to their size. An example of this is the work of Türkben et al. [22]. However, in most cases, the colorimetric evaluation of pseudo-fruits was not conducted for the purpose of assessing their ornamental value; rather, it has been undertaken to examine the quantitative and qualitative characteristics of the plant pigments responsible for their coloration. For instance, Mallick et al. [23] measured total carotenoids, β-carotene, total anthocyanins, and total betacyanins. The biochemistry of pseudo-fruit coloration has also been widely studied; based on these works [24,25,26,27], the coloration is primarily determined by carotenoids, including β-carotene, lycopene, β-cryptoxanthin, rubixanthin, zeaxanthin, and lutein. The publication by Uggla et al. [28] is particularly interesting, in which the ripening process of the rose hips of *Rosa dumalis* Beschst. and *R. rubiginosa* L. species was evaluated using mathematically interpretable CIE color coordinates. This is especially relevant, as our own studies on decorativeness are also based on the CIE (International Commission on Illumination) L*a*b* system and the color standards derived from it. In contrast, Cunja et al. [29] not only recorded the color of rose hips but also examined their red color content. Based on the hue parameter of the CIE L*C*h* system, they identified the reddest samples with a hue value of h* = 17.5°. However, it remains unclear what exactly they meant by “reddest,” as this was not defined. Symoneaux et al. [30] also emphasized the importance of sensory evaluation in the study of ornamental plants. Medveckienė et al. [31], on the other hand, investigated the fruit color of *Rosa canina* L. and *Rosa rugosa* Thunb. species using both the CIE L*a*b* system and the derived CIE L*C*h* chromatic dimensions.

Surprisingly few studies deal with the measurement of the ornamental value of roses. One of the rare examples is the work of Santagostini et al. [32], which primarily demonstrates—in the case of flowers and foliage—that evaluation is not possible without objective measurement. Raju et al. [33], on the other hand, developed an assessment method called the “Ornamental Hip Index” to describe species and cultivars suitable for landscape roses. However, in their work, the Ornamental Hip Index involves a relatively simple calculation and is essentially equivalent to the fruit shape index.

Cultural and historical reasons may explain why the decorative value of roses is specifically associated with flowering, while rose hips are often overlooked, even though they remain visible on the plant for months and can be particularly striking in autumn and winter [34]. For example, Maria et al. [35] examined 13 parameters of ornamental and planting value, yet none of these focused on rose hips. Hip-based decorativeness is also not a criterion in the well-known German ADR (Allgemeine Deutsche Rosenneuheitenprüfung) rose evaluation system, although, according to a 2002 data sheet [36], as many as 10 parameters are assessed. Parsons et al. [37] evaluated ornamental plants using automated image analysis and indexing; however, they also considered only foliage and flowering. The lack of scientific evaluations is all the more striking because horticulture does recognize the aesthetic value of rose hips, and there are even targeted breeding programs for this purpose. Notably, the Patrucco family specializes in breeding rose varieties suitable for dried floral arrangements [38]. What is particularly lacking is an objective method for measuring ornamental value. To our knowledge, the only previous attempt to quantify this trait was described by Boronkay [39].

In order to provide a comprehensive literature review, it also seemed necessary to analyze studies on other ornamental plant species. For example, Gurbanov et al. [40] evaluated coniferous plants, but in assessing pinecone quantity, they distinguished only five categories—as in this group, the fruit is not a determining element of decorativeness. East African aromatic plants from the genera *Calodendrum*, *Gardenia*, *Erica*, *Jasminum*, *Adansonia*, *Afzelia*, and *Sterculia* were evaluated using index formation [41], where aspects of decorativeness were also considered. However, the applied indexing method is difficult to follow due to the publication language being Chinese. Goncharovska et al. [42] studied *Malus* species, but the apple fruits were evaluated only from a pomological perspective. Wang et al. [43], on the other hand, assessed fruit retention versus fruit abscission in ornamental crab apples. Stoenescu et al. [44] examined this specifically from the perspective of ornamental value, but did not construct indices from the pomological data. Interestingly, most studies on fruit-based decorativeness have been conducted on *Malus* (crab-apple) species, and according to Ikase [45], there is even a breeding program for this purpose in Lithuania. More generally, Wang [46] examined the color of ornamental plant species and the harmony between plant and environmental colors, using the Natural Colour System (NCS), which is based on the color opponency of human vision and similar to the CIE systems we use. Guo et al. [47] investigated correlations between fruit coloration and carotenoid content and, similarly to our own results, identified the CIE a* value as the most useful chromatic parameter. The above overview demonstrates that the Q×Q ornamental value assessment model developed for evaluating cultivar collections is a unique and independent work and does not rely on any previously established international model. Its only precedent is the evaluation system for flowers and foliage based on a similar logic, which was developed by some of the authors of the present study.

### 2.2. Literature Review of Factors Influencing Rose Hip Productivity in Roses

Although rose hip production is strongly related to flower production, which is itself influenced by both rootstock effects and environmental factors, only a limited number of publications have examined this relationship. Scientific studies investigating these factors under open-field conditions in a continental climate are scarce; most available studies have been conducted either under subtropical climatic conditions or in greenhouses. For example, Niu et al. investigated the drought tolerance of different rootstocks, but the rootstocks included in their study are frost-sensitive and are intended primarily for greenhouse production [48]. Although Rowley et al. conducted their study under open-field conditions, it was performed in a subtropical climate. Therefore, the direct applicability of their results to continental climatic conditions is limited [49]. Balaj et al. included flower production among the traits evaluated in their study of rootstock effects. However, they investigated the compatibility of only one rootstock cultivar, *Rosa corymbifera* Borkh. ‘Laxa’. Although flower number was assessed, detailed quantitative results were reported only for the flower diameter [50].

Studies investigating flower production and environmental effects have likewise been conducted predominantly under greenhouse conditions. An interesting exception is the study by Liang et al., who investigated the relationship between heat stress and, among other traits, flower production. They reported that even a short period of severe heat stress significantly reduced flower production, with 44 °C used as the heat-stress treatment [51]. The authors of the present manuscript likewise observed reduced flower production and a poor fruit set. However, while all of the study years were characterized by summer heat events in the place of the evaluation, temperatures remained well below the 44 °C applied in the heat-stress experiment of Liang et al. Therefore, a direct comparison between the two studies is difficult.

The influence of soil conditions should also be considered, as it affects flower production and, indirectly, rose hip production. For example, Anzu-Man-Ara et al. examined this effect in container-grown roses [52]. However, the relevance of their findings is largely confined to artificial growing media, whereas, in open-field rose collections, the natural variation in soil properties is generally far less pronounced than the differences among the substrates used in controlled experiments.

Barry et al. addressed this question specifically in relation to rose hips; however, their study involved North American wild rose species rather than garden roses. Among the factors examined, fertilization had the most pronounced effect on rose hip production. In contrast, soil surface management had only a moderate effect on the rose hip weight, whereas grassed inter-rows were associated with reduced fruit yields [53].

The role of soil has been investigated in both open-field cultivation and controlled growing media. Most studies have focused on soil salinity, as roses are considered salt-sensitive plants. Salinity is primarily a concern in artificial growing media, whereas under open-field conditions, it is generally problematic only in coastal regions. According to published studies, electrical conductivity (EC) values of approximately 2.24 dS m^−1^ during bud break and 4.48 dS m^−1^ during the growing season represent the threshold for salt tolerance in roses [54]. Massa et al. [55] reported that high NaCl salinity (up to 63 mol/m^3^) primarily impaired nitrate (NO_3_^−^) uptake in roses.

However, studies on saline (Solonchak and Solonetz) soils are scarce, despite the fact that soil salinization is expected to become an increasingly important challenge for rose cultivation under climate change. One notable exception is the study by Akzhunis et al. [56], who also evaluated garden roses in Kazakhstan. Their results showed that garden rose cultivars exhibited responses to heat stress and soil salinity similar to those observed in broadleaf trees, although the variability of physiological parameters—including the transpiration rate, leaf water content, chlorophyll a and b content, and leaf carotenoid content—was considerably lower in roses.

### 2.3. Literature Review of Q×Q Model

The development of the Q×Q model began in 2006 [57], when we sought a method to demonstrate the relationship between visual scoring (ranking) and the total fresh leaf mass of rose plants. This was necessary because, although leaf density can be measured as leaf mass, collecting the leaves severely damages the plant and is extremely time-consuming. At this stage, we first demonstrated that the relationship between the class numbering described in the text but characterized by quantitative production values, and the calculated production value characteristic of a given class is not linear, but rather follows a power or exponential pattern. A year later [58], based on the developed quantitative index, we were already able to evaluate several bedding rose cultivars. However, since foliage quality as a factor of decorativeness was not interpreted, the logic of the full model based on the product of quantity and quality was published only in 2009 [59] on the complex evaluation of flowering. The applicability of this approach to modeling fruit-based decorativeness, however, was first published in 2018 [39].

### 2.4. Literature Review of Color Conversion Centre

For the colorimetric measurement of rose hip ripening, the identification of outliers, and the analysis of color variegation in rose hips, it is necessary to use the extremely sophisticated CIEDE2000 (or CIE ΔE_2000_) standard [60], which calculates non-linear distances between colors. Such analyses were previously not feasible for large datasets due to the lack of suitable software. For this reason, we developed a program called “Colour Conversion Centre” [61], which is freeware. Although the reliability of the software cannot be formally validated through peer review, it has been used in numerous scientific studies. Among these, in the past three years, the following works have cited—and thereby validated—the program: Nastiti et al. [62] evaluated chicken meat, Taylor et al. [63] used the program for the visual analysis of porphyrins, Dremelj et al. [64] applied it in the dendrochronological evaluation of *Abies alba*, Vilcapoma et al. [65] used it to determine vitamin content in *Physalis peruviana*, Novikov et al. [66] employed it for color referencing in herbarium digitization, and Cutajar et al. [67] evaluated painted surfaces using the CIEDE2000 colorimetric difference calculations provided by the Colour Conversion Centre.

## 3. Results

### 3.1. Brief Summary of the Results Following the Outline Presented in the “Introduction”

(A) Development of a Quantity Index (Figure 1)

(1) The estimated production of rose hips for 131 items was classified (C_hip_), using 15 subclasses (0–7 main classes, with 0.5 steps intervals).
(2) The visible surface area of 430 rose hips was measured and calculated based on the length and maximum width of the pseudo-fruits, modeled as the surface area of an ellipse (A_hip_).
(3) For the 131 items, the number of rose hips per unit shrub area (N_hip_) was recorded.
(4) Based on steps (2) and (3), the production value (P_hip_) was calculated for the 131 items.
(5) Regression analysis was performed between C_hip_ and P_hip_ values. Both the model and the parameters were significant at *p* < 0.001, with an R^2^ = 0.92 determination coefficient. The function is a power type: Y = 0.12792 × X^3.00120^, where Y = P_hip_, X = C_hip_
(6) According to the function calculated in step (5), the Quantity Index is the normalized P_hip_ where X_min_ = 0 and X_max_ = 43.98.

(B) Development of a Quality Index (Figure 2)

(7) Under laboratory conditions, 22 evaluators scored 21 rose hip samples for ornamental value.
(8) Using factor analysis with Varimax rotation, unusual evaluations were identified for two evaluators, and their scores could not be validated, so the work of 20 evaluators was accepted.
(9) Instrumental color measurements were performed on the rose hips evaluated in step (8). The calculated parameters included the following: CIE L*a*b*, CIEDE2000, and unprocessed reflectance values.
(10) Good correlations were found between the scored aesthetical value (step 7) and measured color parameters (step 9). At a significance level of *p* < 0.001, the strongest correlation was observed for the CIE a* parameter, with an R = 0.95 determination coefficient.
(11) Based on step (10), the Quality Index = normalized CIE a* value, where X_min_ = −11.70 and X_max_ = 46.04.

(C) According to steps (5) and (10), we consider that the Q×Q model is acceptable, can be calculated, and meets the preliminary expectations.
(D) Application of the Q×Q Model (Figure 3): in 3 years, visual assessments were carried out on 3168 samples, and 30,345 color measurements were performed on 1459 samples. In total, it was possible to calculate OV_hip_ = Quantity Index × Quality Index in 1419 cases, representing 704 items (cultivars and taxa). Among these, 214 varieties showed non-zero ornamental value in all three years.

### 3.2. Detailed Description

#### 3.2.1. Quantity Index (Figure 1)

##### Estimated and Calculated Production Value

In the case of rose hips, the quantitative value was defined as the total visible surface area of pseudo-fruits per unit shrub area; this is the rose hip production, P_hip_. However, as large-scale data collection, this variable could only be estimated and was therefore classified into 15 classes (a 7-class scale with 0.5 increments). The 7-category classification system was established on the basis of preliminary test assessments. Data collection initially employed 5 categories together with a sixth category representing exceptionally high rose hip coverage (production) values. However, it became apparent that the variability in rose hip coverage was greater than expected, requiring an increase in the number of classes.

As a result, the production of the items can only be characterized by their class serial number (C_hip_) in which class it was assigned. Accordingly C_hip_ can take the following values: 0 to 7 with a 0.5 step size. In order to replace the C_hip_ class numbering with real production values, both measurements and ranking were carried out on a sample of 131 items over a period of two years. For the measurements, varieties were selected so that the sample would be as representative as possible and that all ranking classes would be included. We assumed that the shape of rose hips is close to a spheroid; therefore, their visible surface area can be well approximated by the surface area of the longitudinal section of the pseudo-fruit (A_hip_), which is a 2D planar ellipse. Accordingly, the total visible surface area per bush area was calculated using the formula detailed in Section 5: P_hip_ = N_hip_ × a_hip_ × b_hip_ × π × 100/A_bush_. Accordingly, only the size and number of the rose hips were considered in the calculation of P_hip_, while overlap between rose hips was excluded because it appeared negligible, in contrast to our previous work on flower petals [68] After calculating P_hip_ values for 131 items, the following statistical characteristics were obtained: the average is 7.08%, and the median is 3.72%, while the maximal calculated production is 39.24%.

##### Examination of Correlations

**Figure 4:**
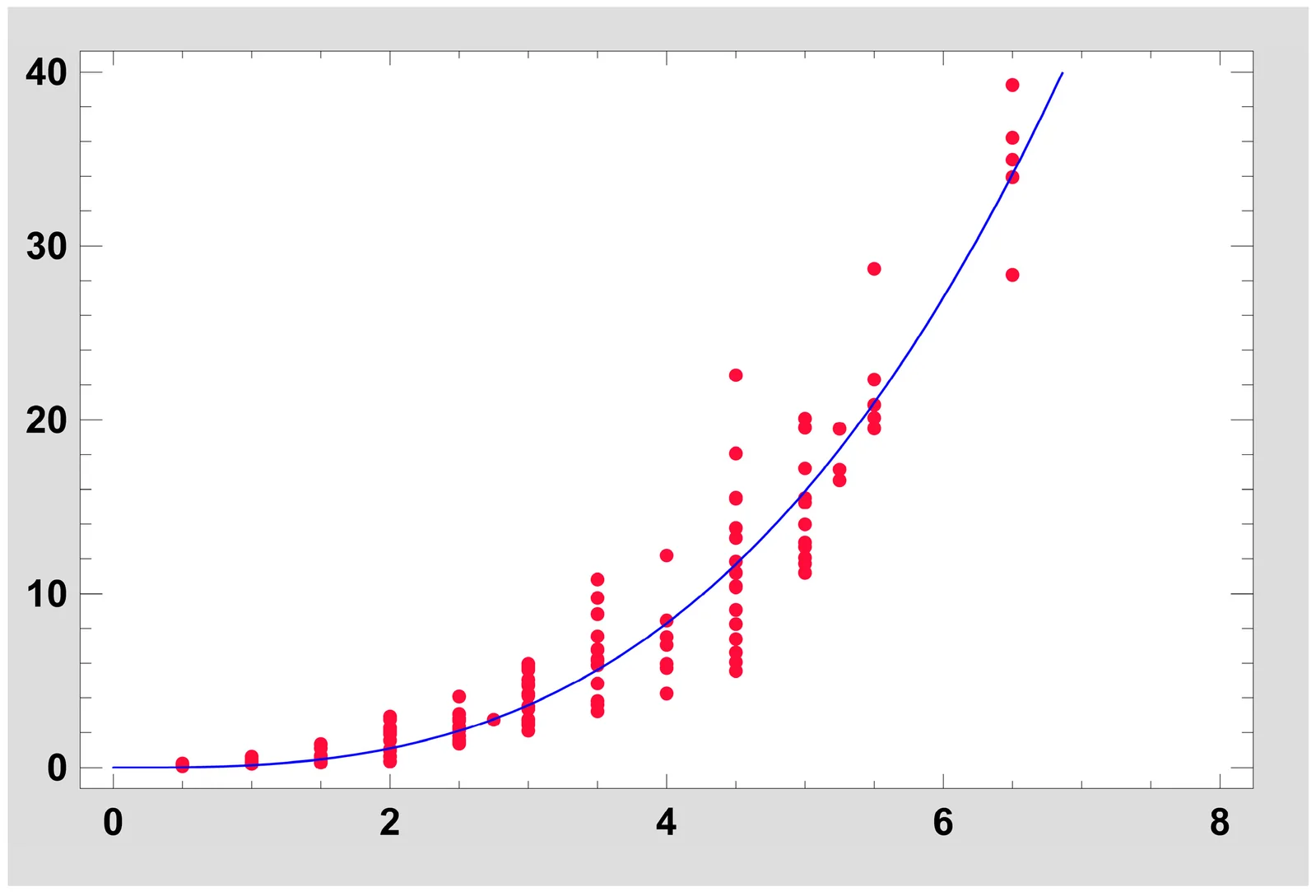
The figure illustrates the relationship between measured (Y) and estimated (X) rose hip production (C_hip_ and P_hip_) in the representative sample. The relationship is evidently of a power-law form. The curve has an R^2^ = 0.92 fit and Y = 0.12792 × X^3.00120^ formula.

In studies on flower and foliage production, exponential or power-type correlations were found between the class numbering (C_hip_) and their corresponding calculated production (P_hip_) [57,68]. Based on this, we assumed that a similar pattern would be observed in this case as well. The relationship between estimated quantity and calculated production of the representative sample was determined via regression analysis (X = C_hip_, Y = P_hip_). Linear, exponential, and power models were tested, as these functions do not produce local maxima or minima. A strong power-type relationship (Y = aX^b^) was found, with a coefficient of determination of R^2^ = 0.92. Both the model and its two parameters (a and b) were statistically significant (*p* < 0.001). The exponential relationship also showed an acceptable correlation; however, it could only be examined as a single-parameter model (a), and in this case, the condition Y = 0 when X = 0 was not met. The linear model was calculated only for its simplicity and interpretability, but the relationship between X and Y did not visually conform to linearity, and its coefficient of determination was significantly lower (R^2^ = 0.575) than that of the power or exponential model. Moreover, for X = 0, the model gives Y = −7.4; therefore, the linear model was rejected. Based on the coefficient of determination, the reliability of the model, and the logical consistency of the correlation, the power function was accepted. Consequently, the C_hip_ class numbering could be substituted with the calculated P_hip_ production values. The parameters of the power function (Y = aX^b^) calculated via regression analysis are a = 0.12792 (*p* < 0.001) and b = 3.00120 (*p* < 0.001). Thus, the exact form of the function is P_hip_ = 0.12792 × C_hip_^3.00120^ (Figure 4). Based on this, Table 1 (last column) presents the production values calculated from the class numbering. The theoretical maximum production (P_hip_ = 100%) would correspond to approximately the 9th class (more precisely C_hip_ = 9.2) according to the power type relationship; however, no examples of rose hip production were found above class 6.5.

Based on the coefficient of determination and the probability of the power-type regression model, the P_hip_ value calculated from C_hip_ values is considered to provide a good estimation of the quantity of a rose hip and, after normalization, to be suitable as the Quantity Index.

#### 3.2.2. Quality Index (Figure 2)

##### Evaluation of Aesthetical Value of an Independent Rose Hip

In order to demonstrate the relationship between the aesthetic value of rose hips and their epicarp color, it was necessary to identify a measurable colorimetric parameter that correlates well with subjective perception of attractiveness. To achieve this, under controlled conditions, a scoring-based evaluation of the aesthetic value of rose hips and colorimetric measurements of the same samples were needed. The total number of evaluators was 22, all participating on a voluntary basis. The evaluation targeted several parameters, of which two are relevant for the present publication: (1) subjective decorative value considering only the epicarp color of the hypanthium, and (2) the same assessment combined with the visual effect of the texture of the hypanthium epicarp, the pedicel and sepals. A total of 21 samples representing characteristic ripening stages were evaluated. Each sample, placed in baskets, consisted of 25–35 water-cleaned rose hips. A scoring scale ranging from 0 to 20 was used for the laboratory assessment of subjective aesthetic value, corresponding to 21 classes. Among these classes, only the two extremes were predefined: 0 = rose hips lacking any coloration and barely noticeable on the plant, 20 = exceptionally showy rose hips.

**Figure 5:**
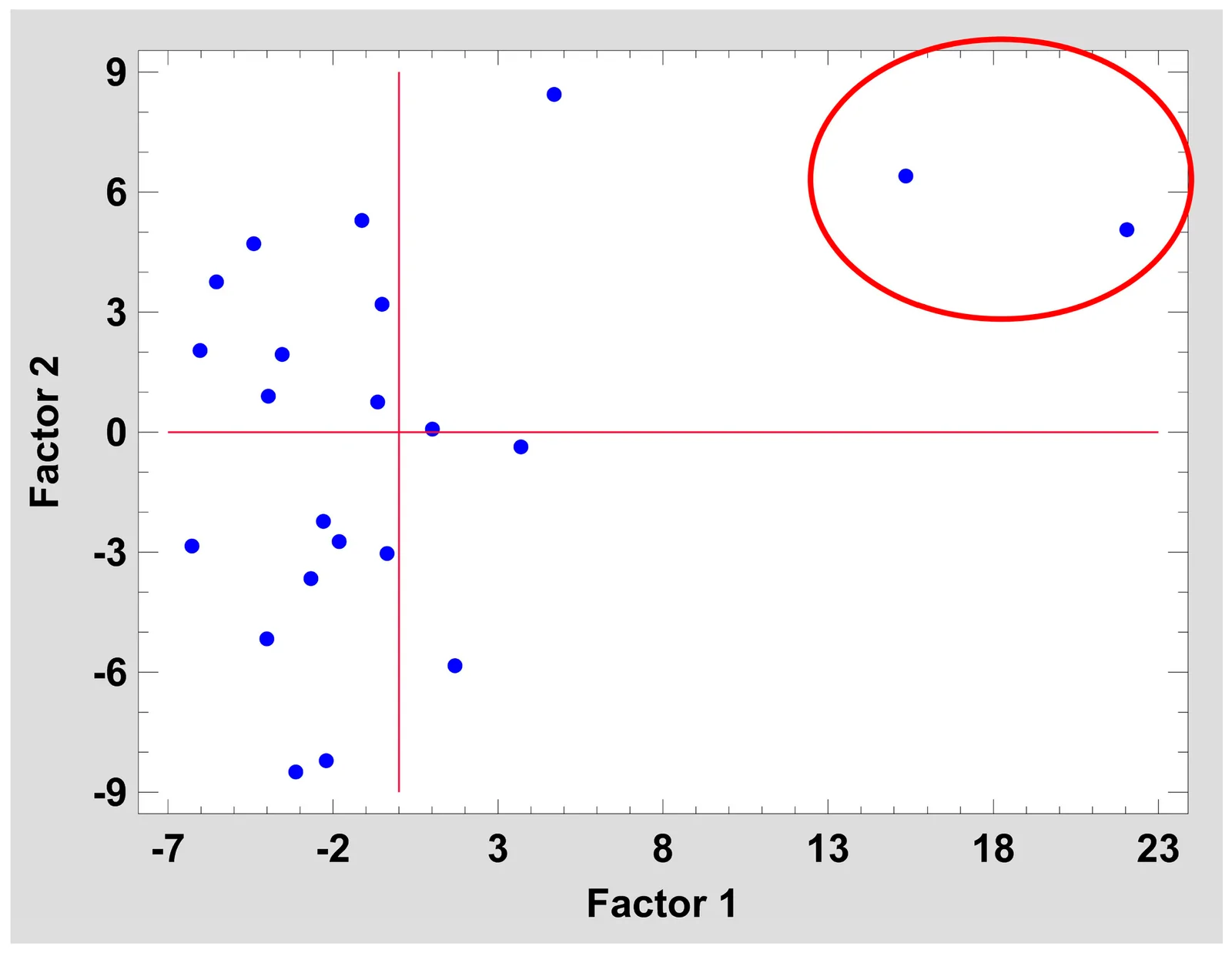
Comparison of laboratory evaluations of rose hip ornamental value by 22 assessors based on three aspects of decorativeness. The results are illustrated based on a factor analysis with Varimax rotation. Each dot represents one evaluator. Factor 1 > 10 values presumably indicate a misunderstood task. Blue dot: 2-dimensional representation of a reviewer’s rating using factor analysis. Values within the red circle reflect extreme opinions, these data were excluded.

To facilitate the evaluation, sample No. 18 (‘Mrs Aaron Ward, Climbing’), considered the least decorative, was fixed at a score of 1. Ideally, a score of 0 would have been chosen as the reference point; however, none of the available samples met this criterion. The scores given by the 22 evaluators were analyzed using Pearson linear correlation and factor analysis (Figure 5) to examine the variability of evaluators’ preferences. Based on this analysis, the ratings of 20 evaluators were found to be mutually consistent, whereas the evaluations of two assessors either did not correlate with or showed negative correlations with the others. Since their evaluations differed fundamentally from those of the rest, their datasets were excluded as results of incorrectly interpreted evaluation criteria. The pattern of the remaining 20 evaluations was more or less similar, with standard deviations ranging between 0.97 < σ < 1.73 across classes. The rose hip samples used in the evaluation and the average scores of the visual assessments are presented in Table 2. These average scores were considered to represent the subjective aesthetic value of coloration, and a colorimetric parameter was sought that correlates well with this value and can be objectively measured.

##### Laboratory Color Measurement of Rose Hips

Color measurements were also carried out on the same samples using a spectrocolorimeter under the settings detailed in Section 5. The parameters measured by the instrument were as follows: CIE L*a*b* standard color dimensions and the unprocessed reflectance values at 40 wavelength ranges between 400 and 700 nm. From these data, CIE L*C*h_33_* (see Section 5) polar coordinate color dimensions were calculated, and the colorimetric distance from the completely unripe rose hip (sample 18: ‘Mrs Aaron Ward, Climbing’) was determined according to the non-linear CIEDE2000 standard, expressed in ΔE00 units. Since the reference value corresponds to the green epicarp color determined based on chlorophyll, higher ΔE00 values indicate more vivid coloration and more intense orange–red pigmentation.

##### Identification of the Best Colorimetric Parameter Describing the Aesthetical Value of a Rose Hip

A total of 392 color measurements were performed. Linear Pearson correlations were calculated between the measured color parameters and the scored aesthetic rating (Table 3). With an exceptionally high coefficient of determination (R = 0.957), the CIE a* parameter showed the strongest correlation. This parameter represents the absolute magnitude of the red component of a color in the CIE L*a*b* color system. In addition, only two other color parameters—both related to CIE a*—reached correlation R > 0.9: CIE h* and the CIEDE2000 color difference from green rose hips, although with slightly lower correlation coefficients. For the strong correlations, significance levels were also tested (*p* < 0.001 in all cases); therefore, these results were accepted. Due to the very high R value, it was concluded that the ornamental value of rose hips can be effectively expressed by the CIE a* parameter of their measured color, which is a one-dimensional and well-measurable parameter. Given the very strong linear correlation, there was no need to explore non-linear relationships, as it seemed unlikely that a significantly better fit could be achieved, and non-linearity was not logically justified either. It was therefore accepted that the Quality Index can be represented by the CIE a* value of the epicarp color of rose hips, as it significantly and reliably reflects the aesthetic preferences of the evaluators.

The question arose as to whether the epicarp color of rose hips alone is sufficient to describe their ornamental value. Therefore, evaluators not only scored the ornamental value of the epicarp of the hypanthium, but also assessed the overall ornamental value of the entire rose hip (including sepal remnants, the pedicel of the pseudo-fruit, and the epicarp texture of the fruit). These two independent scorings were compared using Pearson correlation analysis. Based on the data from 20 evaluators, a relationship of R^2^ = 0.778 was found at the *p* < 0.001 probability level. This indicates that the color of the hypanthium accounts for nearly 80% of the ornamental value of rose hips.

#### 3.2.3. Normalization

The role of normalization is to balance the weights of the variables; as a result, the normalized values fall between 0 and 1. For this purpose, the theoretical extreme values must be specified. In the case of quality, the minimum and maximum values of the measured CIE a* were used: X_min_ = −11.7 and X_max_ = 46.045. In the case of the production (P_hip_), X_min_ = 0 and X_max_ = 43.979 were used, as production values of the class 0 and 7. Using higher class numbering was not considered realistic, as no examples were observed for ranking classes of 7 or above.

### 3.3. Using the Q×Q Model for Evaluating a Rose Collection

Over a three-year period in the Budatétény Rose Garden, we were able to measure the epicarp color of rose hips and simultaneously assess the quantity of visible rose hips throughout the entire collection (Figure 3). Due to high spring and summer temperatures, fruit set was poor; prolonged heat waves, in particular, caused fertilization disorders. As a result, in certain years, many varieties produced no evaluable quantity of rose hips; therefore, 0 pseudo-fruits per bush were common. These samples were excluded from the evaluation. After exclusions, colorimetric data were obtained for 542 samples in 2017, 419 in 2018, and 499 in 2022. For each measured sample, rose hip production was also assessed, allowing both the Quality Index (CIE a*) and Quantity Index (P_hip_) to be determined.

**Figure 6:**
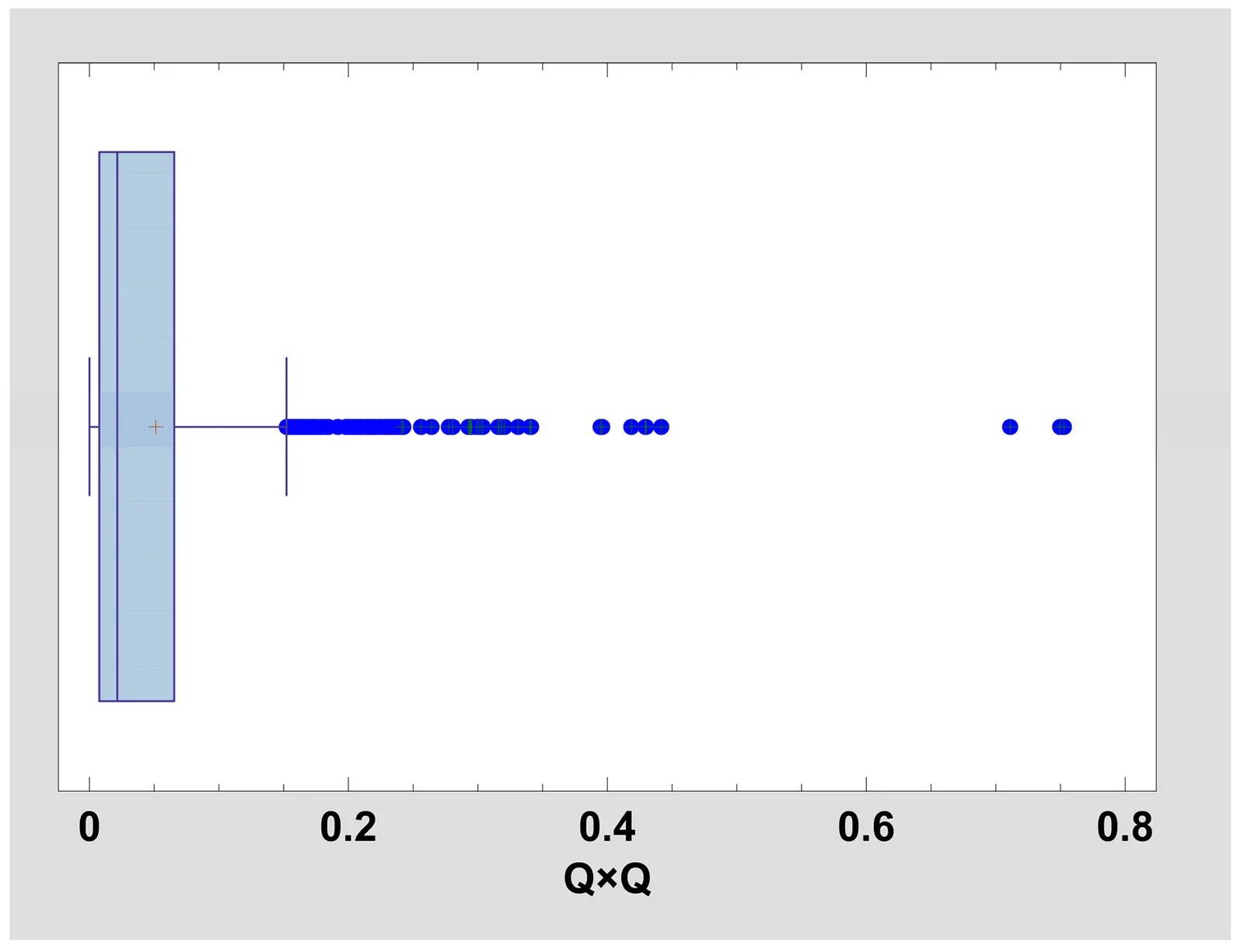
Box and Whisker Plot illustrating the distribution of all hip-based ornamental value (OV_hip_) data obtained in this study. The representation based on the median and quartiles clearly shows that the distribution of OV_hip_ deviates markedly from a normal distribution.

**Figure 7:**
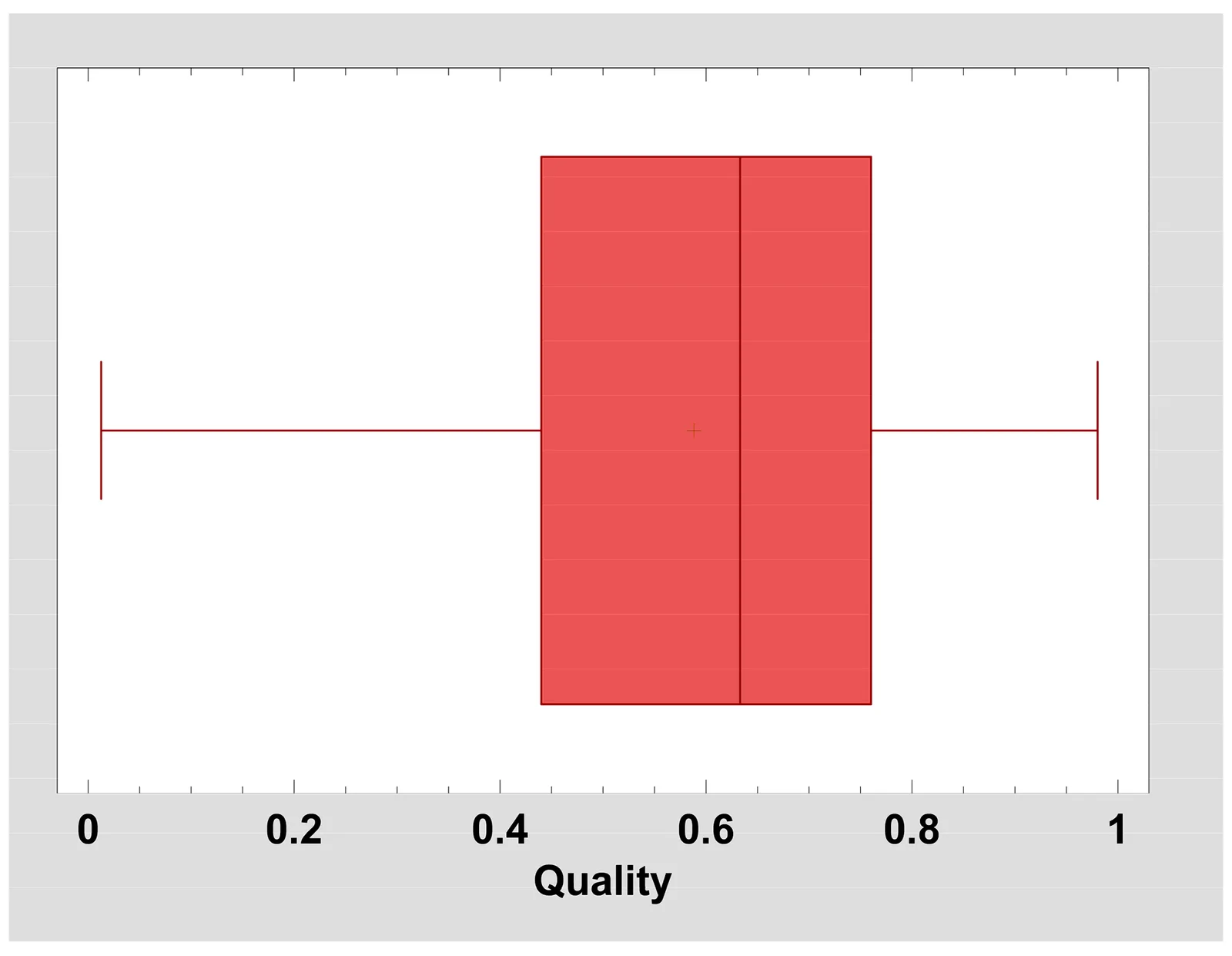
Box and Whisker Plot illustrating the distribution of all Quality Index data obtained in this study. The median- and quartile-based representation shows the relatively balanced distribution of the indices derived from rose hip epicarp color.

**Figure 8:**
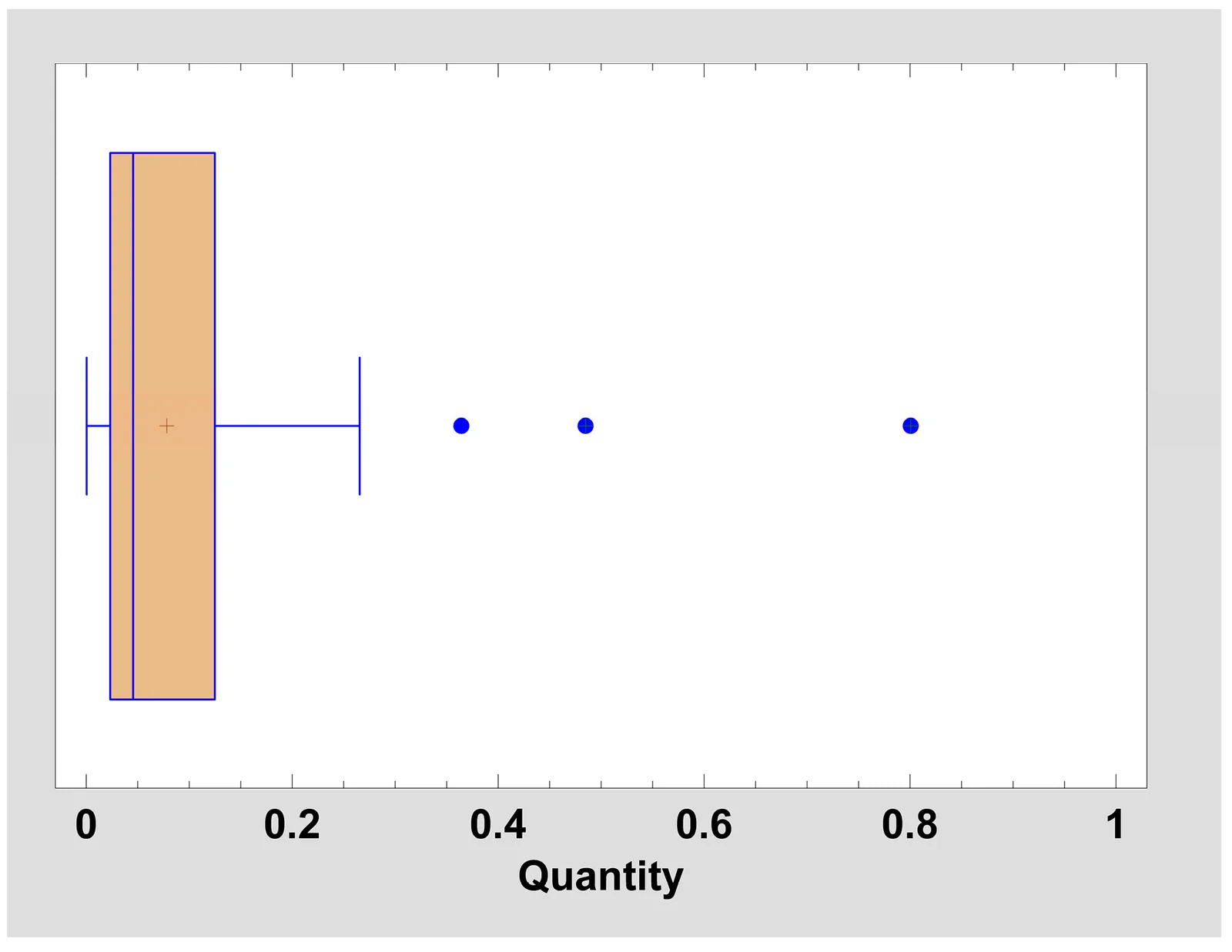
Box and Whisker Plot illustrating the distribution of all Quantity Index data obtained in this study. The median- and quartile-based representation clearly shows that the distribution of the indices based on rose hip production deviates markedly from a normal distribution.

A total of 704 items (taxa and cultivars) were evaluated, representing 1419 assessed samples over the three-year period. Table 4 summarizes the results by presenting the 25 varieties with the highest average OV_hip_ based on three-year data, while Table 5 shows the 25 varieties with the highest single-year OV_hip_. In many cases, for many items, only one or two years of data were available, as no rose hips were present on the plants in certain years. Altogether, 214 varieties have both quantity and quality data available for all three years. Considering all measured data, the distribution of the data is highly skewed (Figure 6): 87% of the varieties have OV_hip_ < 0.1, indicating that the majority of the items in the Budatétény Rose Garden are not considered decorative when evaluated on the basis of their rose hips. In fact, only one item was found to be outstanding in terms of both the quantity and quality of rose hips, namely the small-flowered sweet briar (*Rosa micrantha* Borrer ex Sm.). However, this seemingly weak overall performance is clearly attributable to the low quantity of rose hips present on the plants. Only three items had a Quantity Index above 0.5 (*Rosa micrantha* Borrer ex Sm., *Rosa canina* L. ‘Inermis’, and ‘Fragezeichen’), while 664 items had values below 0.2. In contrast, when considering the Quality Index calculated from rose hip epicarp color, these numbers were 87 and 40, respectively. Statistically, this is well reflected in the skewness of the data: γ_OV_ = 3.46, γ_Quantity_ = 2.46, and γ_Quality_ = −0.62 (Figure 6, Figure 7 and Figure 8).

The year-to-year stability of hip-based decorativeness of the rose collection was found to be weak. Only those samples for which data were available from all three years (2017, 2018, 2022) were analyzed. Using Pearson correlation analysis, the correlation coefficient (R) for the Quality Index ranged between 0.366 and 0.471; for the Quantity Index, it ranged between 0.577 and 0.618; and for the overall ornamental value, it ranged between 0.447 and 0.472. In all cases, the significance level was *p* < 0.001 (Table 6). One-way ANOVA was applied to determine whether a year effect could be detected in the data. With *p* < 0.05, the existence of such an effect was confirmed. According to the Tukey HSD post hoc test, for the Quality Index, the year 2018 was significantly different from the others; for the Quantity Index, the year 2017 was distinct; and for the overall ornamental value (of fruiting plants), 2017 also showed a characteristic difference from the other years. In all three cases, the remaining two years could not be statistically distinguished from each other (Table 7).

In addition, the OV values were also averaged for each cultivar group (Table 8). The lowest value was OV_hip_ = 0.004 for moss roses, while the highest was 0.297 for wild rose species. Among the cultivated rose groups, the highest value was OV_hip_ = 0.223 for lucieae (formerly wichuraiana) hybrids.

## 4. Discussion

### 4.1. Comparing the Results

Only a limited number of similar studies have been published; therefore, comparing our results with those of others is somewhat challenging. Previous research has primarily focused on measuring fruit color, but this has rarely been quantified, and we found no examples of measuring the relative visible surface area of rose hips. Goncharovska et al. [42] categorized ornamental crab-apples based on measured data using the unweighted pair group method with arithmetic mean. This approach follows logic opposite to that of our study, in which categorization served as the starting point. Ning et al. [69] also worked with a cultivar collection, examining domestically bred crab-apple varieties. They evaluated exocarp color, exocarp coloring pattern, and fruit dimensions (vertical and horizontal diameters) and reported relatively low phenotypic homogeneity. In contrast, our results indicated a higher level of homogeneity, exceeding even the year-to-year stability of fruit yield. Fataliyev et al. [70] likewise investigated fruit color in roses using the same CIE L*a*b* standard applied in our study. However, they compared colorimetric values only with nutritional parameters and did not examine the aesthetic value of rose hips.

### 4.2. Evaluation of Q×Q Model

The authors’ objective was to develop a method capable of evaluating extensive cultivar collections even when only a very short time is available. Based on our results, it can be concluded that the Quantity Index × Quality Index (Q×Q) method is well suited for assessing the hip-based decorativeness of garden roses and meets the requirements: huge data-basis, fast data-collection, suitable for mathematical statistics, and strong reliability levels of the applied statistical models.

In the case of the Quantity Index, the main question was whether a relationship could be identified between subjective ranking and actual production and how interpretable this relationship would be. We found a non-linear, power-type relationship between measured rose hip production and estimated production (Figure 5), which was consistent with our hypothesis, as similar relationships had previously been demonstrated for foliage [57] and flower production [68]. The difference between the P_hip_ values of successive classes is not constant, although the textual definition of the classes seems to imply equal class intervals. The difference in production between higher-numbered classes is larger. The strong correlation and the statistical significance of the model support the validity of the method and make it acceptable to calculate production values (P_hip_) from class numbering (C_hip_) using the equation P_hip_ = 0.12792 × C_hip_^3.0012^ for every class, for any item. It is evident that replacing class numbering with the mean production values of the respective classes does not improve the resolution of data collection; however, it substantially increases the accuracy of the index, particularly given the non-linear nature of the relationship.

A further question that arose was how many additional classes would be required in cases of very high production levels that were not observed during data collection. Based on the power function obtained from the regression analysis, the theoretical maximum rose hip production (P_hip_ = 100%) would be reached at subclass C_hip_ = 9.2. In contrast, in the Budatétény Rose Garden, the highest observed class value was 6.5, corresponding to P_hip_ = 27%, that is, approximately 27% of the bush surface area covered by rose hips. Nevertheless, the highest value obtained from the measurements and calculations was 39%, for a sample of *Rosa micrantha* Borrer ex Sm. in 2020. However, it is possible that higher values may occur in plantations specifically established for rose hip production, particularly when cultivars bred explicitly for high fruit yield are used.

According to our preliminary hypothesis, the ornamental value of a rose hip is determined—besides its quantity—by the color of the epicarp of the pseudo-fruit, while its other visible characteristics have a negligible effect. Based on the scoring evaluation and colorimetric measurements, this hypothesis was confirmed with an adequate level of reliability. This indicates that the ornamental value of rose hips is determined by their color to nearly 80%, provided that they are examined on the plant under open-field conditions. In dried floral arrangements, however, the relationship may be quite different, as the role of green pseudo-fruits and large sepals presumably becomes more significant. The above statement is supported by a similar result obtained in Brazil during the evaluation of ornamental chili pepper varieties [71]. There, a large variation was found in the opinions of evaluators, but fruit shape was not relevant to personal preference. In contrast, fruit coloration proved to be much more significant, although it was not quantified in the study. Interestingly, studies by Kouassi et al. show the opposite for the ornamental value of the fruit of *Malus domestica* [72], where a strong correlation was found only with fruit size, while the relationship between fruit color and ornamental value was particularly weak. However, it should also be taken into account that apples are much less vividly colored, but larger, than peppers or rose hips. It is therefore not surprising that in ornamental peppers [73], the colorful fruit epicarp was found to be a factor that significantly increases the ornamental value; however, fruit color was not included in the variance analysis of the study.

We also needed to demonstrate that there exists a colorimetric parameter by which the seemingly subjective aesthetic value of a fruit can be described accurately. When compared with scoring evaluations by assessors, three color parameters showed high correlations, which is not surprising, as these are interrelated. CIE a* describes the red–green component of a color, based on the concept that these are mutually exclusive factors. The CIE h* (and h_33_*) hue angle measures the same along a circular arc, while the CIEDE2000 color difference was calculated from the unripe green rose hip. Thus, this ΔE_00_ color distance is also proportional to the coloration of the rose hip. In the case of CIE a*, the calculated correlation value appears outstanding, making it an ideal parameter for measuring the ornamental value of rose hips. This is indirectly supported by Turkish studies [74]; they found that during the ripening of wild rose hips, the yellow component (CIE b*) and lightness (CIE L*) decrease, while the red component (CIE a*) increases. An additional advantage of the CIE a* parameter is that it is measured directly by the spectrocolorimeter, and its use does not require any data-transforming calculations that could distort the data. Accordingly, the ornamental value of fruiting garden roses has become measurable and calculable. Supporting our hypothesis, the aesthetic value of rose hips (as a Quality Index) can be measured, while rose hip production (as a Quantity Index) can be estimated, and all of this is supported by mathematical statistics.

### 4.3. The Hip-Based Ornamental Value of the Collection of the Budatétény Rose Garden According to the Q×Q Model

In the Budatétény Rose Garden, we were able to examine a large number of roses: for 704 items (cultivars and botanical taxa), at least one year could be evaluated, and in 214 cases, data were available for all studied years. However, two aspects must be taken into account when evaluating the data. First, the surveyed rose garden primarily contains hybrid tea roses and floribundas, while landscape and climbing roses are present only in negligible numbers. Second, many groundcover, landscape, and polyantha roses could not be evaluated, because the diameter of their rose hips did not reach 8 mm, which corresponds to the size of the measuring aperture head of the spectrocolorimeter used for color measurements. Thus, roses with very small rose hips could not be included in the measurements, even if the number of their pseudo-fruits was often very high.

We found many items with low ornamental value, and in practice, only a single item proved to be outstanding: *Rosa micrantha* Borrer ex Sm., originally planted as a rootstock. This seemingly weak performance clearly results from the quantity of rose hips. In contrast, when considering the Quality Index calculated from the epicarp color of the rose hips, the values are much more evenly distributed (Figure 6, Figure 7 and Figure 8). Based on the data, the differences between cultivars were strikingly large, making further mathematical analysis of this aspect unnecessary. However, the question arose as to whether a year effect could be detected, since the year-to-year stability of the ornamental value of fruiting plants was found to be weak among cultivars. This was primarily due to the annual fluctuations in rose hip production. Nevertheless, variance analysis demonstrated that differences in weather conditions between years have a statistically significant effect on the ornamental value of fruiting roses.

When examining ornamental values by cultivar groups (Table 8), considerable differences can be observed. From the perspective of hip-based ornamental value, wild species and their cultivars proved to be the most valuable. In these cases, not only the quantity of fruits but also fruit coloration is excellent; it is therefore no coincidence that this particular group is collected for medicinal purposes [19]. The next most decorative group is the so-called Wichuraiana hybrids, which are hybrids of *Rosa lucieae* Franch. & Rochebr. (formerly *R. wichuraiana* Crép. ex Déségl.). Similarly outstanding is the Kordesii group, a hybrid complex derived from *Rosa rugosa* Thunb. × *Rosa lucieae* Franch. & Rochebr. Both groups consist of climbing-type cultivars and landscape roses.

Hybrid tea roses are also worth mentioning, as they are perhaps the most popular group of roses worldwide. However, this group—specifically bred for cut flower production—has particularly poor ornamental value of fruiting plants. According to our evaluation, neither the quantity nor the quality of their rose hips is sufficient for them to be considered decorative in winter. The rose hips of hybrid teas often fall off early, or if they persist, they tend to be corky in texture and dull in color. The overall appearance is only slightly improved by the fact that rose hips of hybrid teas are often quite large, because—at least under Hungarian conditions—they do not develop strong coloration and generally remain brownish–green. The best hybrid tea cultivar was found to be DELamo (Delbard, 2004), with a value of OV_hip_ = 0.22, while the best floribunda (bedding rose) was ‘Méphisto’ (Mallerin, 1951), with an OV_hip_ value of 0.28 (Table 8). All this clearly indicates that cut and bedding roses selected primarily for their flowers have minimal rose hip production, and if one wishes to plant roses that remain visually attractive in winter, it is advisable to choose less floriferous landscape varieties, landraces, and wild roses.

A related practical question is whether developing rose hips should be removed. Wang et al. conducted a comprehensive study demonstrating that dead-heading did not significantly accelerate repeat flowering in shrub rose cultivars. Consequently, retaining rose hips on the plant may be a reasonable management option [75].

### 4.4. Possibilities for Further Development

The main limitation of the Q×Q model lies in the resolution of the estimation of rose hip production, that is, in the number of ranking classes. Therefore, our method can primarily be improved by increasing the number of classes or—when examining a smaller number of items—by directly measuring rose hip production eliminating estimation altogether. In addition, the accuracy of evaluating the ornamental value of fruiting cultivars clearly depends on the number of repetitions of measured and estimated data, as well as on the number of evaluators involved in the assessment. However, since the Q×Q method was specifically developed for rapid data collection in large populations, the number of repetitions cannot be increased indefinitely.

## 5. Materials and Methods

### 5.1. Indices

OV_hip_: ornamental value of a fruiting rose bush based on the Q×Q model; P_hip_: calculated rose hip production (visible rose hip surface area per bush surface area); C_hip_: class numbering of the estimation of rose hip production by ranking; A_hip_: measured surface area of a rose hip; A_bush_: measured surface area of a rose bush, excluding the basal part without fruits and foliage; N_hip_: number of rose hips.

### 5.2. Terminology

“ornamental value of rose hip”: decorativeness of one piece of rose pseudo-fruit; “ornamental value of fruiting plant”: decorativeness of a fruit-bearing bush; “class numbering”: the sequence numbers of the ranking classes, starting from 0. In theory, this value can take only integer values, but to refine the estimation during field data collection, step intervals smaller than 1 may also be used, typically half-step increments: 0, 0.5, 1, 1.5, etc.

### 5.3. Location

Both the quantitative and qualitative parameters of rose hips were recorded in the Budatétény Rose Garden (southern Budapest, Hungary), as this was the only site in Hungary where a sufficient number of rose varieties bearing pseudo-fruits was available. Furthermore, since it is not a private collection, continuous measurements could be carried out.

### 5.4. Dates

Rankings were conducted on 8 November 2017 (1484 items), 6–7 November 2018 (1085 items), and 12 October 2022 (599 items). The laboratory determination of rose hip color was carried out on 28 November 2016 (392 datasets), while open field instrumental color measurements were performed on 10–19 November 2017 (419 items, 11,220 measurements), 5–12 November 2018 (541 items, 8965 measurements), and 3–17 October 2022 (499 items, 10,160 measurements). Measurements required for calculating P_hip_ values were conducted between 10 and 18 October 2015 (1344 measured values) and between 29 October and 24 November 2020 (1720 measured values).

### 5.5. Cultivar Names

Since the registered cultivar name is often a code name and not always easily interpretable, in this work, we also use the commercial names recommended for exhibition by the American Rose Society (ARS) [76]. If the registered name is a code, the letter combination referring to the breeder is always capitalized.

### 5.6. Ranking (Classification via Visual Estimation)

We examined cultivars and species that were planted in bed systems over a length of at least 2 linear meters and having a minimum bush surface area of 2 m^2^. The classification was carried out using a 0–7 scale with increments of 0.5, resulting in a total of 15 classes (e.g., 0, 0.5, 1, 1.5, etc.). For the definitions of the classes, see Table 1. Class 7 represents the highest realistically occurring fruit production, rather than the theoretical maximum of 100% hip coverage. No practical examples were found for classes higher than 6.5.

### 5.7. Measurement of Rose Hip Production

A transparent plastic sheet measuring 0.5 × 1 m (0.5 m^2^), marked with a 10 × 10 cm grid, was used for data collection. The number of pseudo-fruits beneath the grid was recorded (N_hip_). For each item, the average length (a_hip_) and diameter (b_hip_) of the rose hips were calculated based on 3 measurements taken from 5 individual pseudo-fruits. From these values, the visible surface area of a rose hip was estimated by modeling the rose hips as planar (2D) ellipses, where the surface area or A_hip_ = a_hip_ × b_hip_ × π. Based on this, the formula of rose hip production was the following: P_hip_ = N_hip_ × a_hip_ × b_hip_ × π × 100/A_bush_. Its dimension is %_A/A_.

### 5.8. Color Measurement

The field instrument used for colorimetric measurements was a Konica-Minolta 600d spectrocolorimeter (Konica Minolta, Inc., Tokyo, Japan). The measurement parameters were as follows: D65 illumination (simulated daylight) [77] and a 10° standard observer [78] with diffuse 8° SCE (glare-free) measurement. The latter parameter is an instrument setting and not an optical standard [79]. The recorded colorimetric data included CIE L*, a*, b*, C*, and h* values [80], as well as the instrument’s unprocessed reflectance values at a 10 nm resolution over the 400–700 nm range. A limiting factor was the size of the pseudo-fruits. Since the instrument’s measuring aperture is 0.8 cm in diameter, items with rose hips smaller than approximately 1 cm in diameter had to be excluded from measurements, even if a large number of fruits were present on the plant. Each rose hip was measured in a “W” pattern with five repetitions to ensure that the entire surface of the pseudo-fruit was assessed. Measurements were performed on 5 rose hips per item; however, fewer were measured if an insufficient number of measurable fruits were available on the bush. No samples were collected from the ground.

### 5.9. Color Dimensions and Color Spaces

The basis of the color measurement was the CIE L*a*b* or CIELab [81] standard provided by the spectrocolorimeter, which, for better interpretability, was converted into the CIE L*C*h* or CIELCh polar coordinate system. Thus, instead of the orthogonal axes of the red–green (CIE a*) and yellow–blue (CIE b*) dimensions, color saturation (CIE C*, or chroma) and hue (CIE h*) were expressed. For describing the color of pseudo-fruits, we used a modified color dimension system developed by some of the authors, the CIE L*C*h_33_*, because, in this system, averaging and interpretation of plant colors are much easier. This method has been published both in English [82] and in the authors’ native language [39]; the modification affects only the description of hue (h*) of the original standard CIE L*C*h* system. The calculation is as follows: h′ = h* − 33°; if h′ > 180°, then h_33_* = h′ − 360°, otherwise h_33_* = h′. As a result of this modification, h_33_* = 0° corresponds to neutral (spectral) red, such as the “19—Scarlet” and “719—Signal Red” colors of the British Colour Council standard [83]. Values above 180° (180° → 359°) are replaced by negative values (−180° → −1°), so that warm colors have positive values and cool colors have negative ones. This eliminates the difficult-to-interpret 360° = 0° discontinuity. However, it creates a −180° = +180° one, though this logical contradiction occurs only in the turquoise region, which is very rare among plant colors. In all other respects, the official CIE h* and the modified h_33_* are identical.

### 5.10. Data Exclusion

Measurements were excluded when the spectrocolorimeter recorded a value of 0 in any wavelength range, which was presumably attributable to measurement-related issues. Items were excluded if no rose hips were present. This could be due to sterility or fruit drop caused by mechanical factors, which could no longer be traced at the time of assessment.

The identification of outliers and the corresponding data exclusion were based on the measured CIE L*C*h_33_* values. Based on our experience and test measurements, the following threshold values were considered anomalous: L* > 70, C* > 90, and h_33_* < −50° or h_33_* > 90°. In the case of L*, extremely low values were not considered anomalous, as desiccation and other biotic factors often cause color darkening, which can be regarded as natural and potentially cultivar-specific. In contrast to similar data-collection approaches [82], the large variance (σ) of the measured values was not considered measurement error and did not lead to data exclusion. This is because extreme color variation within a single pseudo-fruit is common in rose hips, and coloration is often localized.

### 5.11. Mathematical Statistics

Linear correlation and regression analyses were performed using Statgraphics Centurion 18.1.01 software. Non-linear CIEDE2000 standard colorimetric differences (color distances expressed in dimension ΔE_00_) were calculated using the self-developed Colour Conversion Centre, version 4.2g [61], which is based on the works of Luo et al., Sharma et al., and Wee Kheng [84,85,86]. This software consists of Excel spreadsheets and, as freeware, is accessible to everyone. The colorimetric difference calculation can be found on the CIEDE2000 worksheet, where the default parameterization was applied: K_L_:K_C_:K_h_ = 1:1:1 weighting factors.

### 5.12. The Normalization Procedure Applied

The formula used was X′ = (X − X_min_)/(X_max_ − X_min_). Since the CIE a* colorimetric parameter, used as a Quality Index, has no theoretical extreme values, the minimum and maximum values were derived from the measured dataset, resulting in X_min_ = −11.70 and X_max_ = 46.04. In contrast, for P_hip_ as a Quantity Index, theoretical extreme values do exist: X_min_ = 0 and X_max_ = 43.98, corresponding to the production values of class numbering 0 and 7, respectively. In fact the theoretical maximum would be P_hip_ = 100 (%), although it is so unrealistic that it can only be calculated mathematically.

### 5.13. Quantity × Quality Model

As none of the steps in the development of the model were adopted from existing methods but instead follow from the authors’ prior conceptual framework, the model is presented in Section 3.

## 6. Conclusions

If rose hip quantity is estimated according to the classes presented in Table 1, the following procedure should be used to calculate the ornamental value of fruiting plants: (1) the recorded data are C_hip_ as the estimated rose hip quantity grouped into 15 classes and the measured CIE a* as the red component of the hip epicarp color. (2) The calculation is as follows: OV_hip_ = normalized (0.12792 × C_hip_^3.00120^) × normalized (CIE a*). Based on this, we were able to evaluate all measured cultivars in the Budatétény Rose Garden using this Quantity Index × Quality Index model.

Based on a three-year evaluation of 704 rose items, the decorativeness of rose taxa and cultivars can also be assessed on the basis of their rose hips, and significant differences can be observed among items. Nevertheless, the hip-based ornamental value of most traditional garden roses, particularly Hybrid Tea and Floribunda cultivars, is negligible. For this purpose, landscape roses and wild taxa are more suitable for planting. Nevertheless, certain Floribunda cultivars are known to produce a substantial amount of rose hips in autumn in addition to their summer flowering; however, these were not included in the present study. In garden cultivars, the removal of rose hips is therefore not essential, particularly as they may also serve as winter food for birds.

Our work may prove helpful for researchers of botanical collections and breeders who wish to evaluate large plant populations. We have demonstrated that production data obtained through ranking (classifying) can be interpreted numerically, and that color—at least in the case of fruits—serves as a reliable descriptor of decorative value. The ornamental value of a fruit can be calculated based on its quantity, size, and coloration. However, it is also evident that relatively few garden roses possess truly high ornamental value when only the rose hips are taken into account.

## Figures and Tables

**Table 1:** Classes of estimated rose hip production (C_hip_) used in the evaluations, their descriptions, and the corresponding production values (P_hip_) calculated using a power function (Y = aX^b^).

Class Numbering	Descriptions for Pseudo-Fruits of Average Size	Mean of P_hip_ [%_A/A_] (Calculated)
0	There are no rose hips in the entire population	0.000
0.5	A few small-sized or one medium-sized rose hip is visible	0.016
1	Only a few rose hips are visible	0.128
1.5	Only a few small groups of rose hips are visible	0.432
2	Rose hips are present extremely sparsely	1.024
2.5	Rose hips are scattered	2.001
3	Rose hips are sparsely distributed	3.458
3.5	The quantity of rose hips is low, but noticeable	5.493
4	Rose hips are clearly noticeable, mostly arranged in small groups	8.200
4.5	Rose hips are relatively densely distributed	11.678
5	Rose hips are densely distributed, but the foliage beneath is still clearly visible	16.021
6	Rose hips dominate the visual appearance	21.326
6.5	Rose hips are dense; in small patches, they almost cover the foliage	27.690
7	Rose hips are extremely dense; in patches, they cover the foliage	43.978
7.5	Theoretical value calculated from the model *	54.096
8	Theoretical value calculated from the model *	65.657
8.5	Theoretical value calculated from the model *	78.759
9	Theoretical value calculated from the model *	93.498
9.5	Theoretical value calculated from the model *	109.970

* No measured data are available for this category.

**Table 2:** Rose hip samples of the laboratory evaluation, average scores of aesthetic value, and CIE a* color parameters (measured according to the D65/10°/SCE standard) of hypanthium.

Item Number	Registration Name Followed by ARS Trade Name if the Two Differ	Average CIE a* (Measured)	Average Score: Hypanthium Epicarp	Average Score: Full Pseudo-Fruit
1	‘Lichtkönigin Lucia’	21.85	14.75	12.65
2	TANolg; ‘Oregold’	15.41	10.85	12.1
3	‘Mrs Aaron Ward, Climbing’	20.02	12.4	12.55
4	‘Gruss an Freundorf’	35.89	14.35	12.65
5	‘Mrs L. B. Coddington’	6.91	2.95	6.5
6	‘Gruss an Freundorf’	38.58	16.4	14.05
7	*Rosa micrantha* Borrer ex Sm.	35.66	18.4	13.85
8	‘Louise Odier’	8.83	6.45	7.45
9	‘Fragezeichen’	31.24	16.6	12.2
10	KORplunblo; ‘Mandarin Ice’	−1.66	2	7.15
11	KORgosumo; ‘Gelber Enger’	31.73	13.4	13.9
12	‘City of York’	39.21	18.75	15.55
13	‘Louise Odier’	22.12	11.25	10.25
14	‘Anne de Bretagne’	−0.167	3.25	10.1
15	‘White Week-end’	0.32	1.5	4.8
16	‘Anne de Bretagne’	15.60	10.7	14.8
17	‘Anne de Bretagne’	−5.50	3.7	9.65
18	‘Mrs Aaron Ward, Climbing’	−4.60	1	4.25
19	‘Town Crier’	7.07	5.65	8.4
20	‘La Jolla’	12.58	6.3	6.55

**Table 3:** Pearson correlations between the average aesthetic value scores of the epicarp of rose hips and their measured chromatic parameters.

Parameter	Correlation Coefficient (R)	Probability if R > 0.9
CIELAB L*	−0.439	
CIELAB a*	0.957	X (*p* < 0.001)
CIELAB b*	−0.049	
CIELAB C*	0.660	
CIELAB h*	−0.935	X (*p* < 0.001)
CIE ΔE_00_	0.937	X (*p* < 0.001)
400 nm	−0.549	
410 nm	−0.670	
420 nm	−0.739	
430 nm	−0.766	
440 nm	−0.775	
450 nm	−0.787	
460 nm	−0.795	

**Table 4:** Quality and Quantity Indices and measured color parameters (in the CIE L*C*h_33_* system) of the 25 items with the highest OV_hip_; all listed items were evaluated in all three years. Cultivars and taxa marked with an asterisk flower only once during the growing season.

Registration Name Followed by ARS Trade Name if the Two Differ	Quantity Index	Quality Index	OV_hip_	CIE L* Lightness	CIE C* Chroma	CIE h_33_* Hue #
* Rosa micrantha Borrer ex Sm.	0.927	0.510	0.474	41.88	49.88	−1.15
* ‘Fragezeichen’	0.865	0.510	0.444	41.31	44.69	−2.17
‘Sympathie’	0.858	0.412	0.356	43.19	49.88	6.47
‘Méphisto’	0.823	0.339	0.278	48.03	51.37	12.26
‘Rudolph Kluis’	0.839	0.298	0.252	45.26	49.00	8.41
DELamo; ‘Cathedral Bells’	0.762	0.251	0.217	51.23	51.92	19.10
BUCbi; ‘Carefree Beauty’	0.844	0.252	0.211	44.29	48.85	6.60
‘Shepherd’s Delight’	0.735	0.272	0.205	50.13	43.98	26.47
DELstrijor; ‘Alfred Sisley’	0.660	0.305	0.194	47.77	43.41	20.15
‘Cyclamen’	0.818	0.225	0.184	50.46	51.97	13.53
* Rosa villosa L. syn: *R. sancti-andreae* Degen & Trautm.	0.703	0.252	0.176	37.90	34.61	−1.14
POULwei, ’Nina Weibull’	0.880	0.189	0.166	48.54	54.47	11.42
‘Stromboli’ (non-registered)	0.716	0.219	0.163	43.51	41.31	11.06
‘Golden Wings’	0.668	0.246	0.157	47.82	44.31	18.82
‘Santana’	0.812	0.192	0.154	43.79	47.73	9.55
‘Kovászna’ (non-registered)	0.839	0.177	0.151	45.45	48.43	7.43
‘Parkdirektor Riggers’	0.791	0.192	0.150	46.33	45.78	9.19
‘János vitéz’ (non-registered)	0.885	0.166	0.148	44.10	50.80	5.48
‘Wision’; (unknown registration name)	0.784	0.192	0.148	48.95	52.46	16.40
‘Concertino’	0.518	0.272	0.143	43.93	30.15	20.82
‘Leonie’	0.683	0.218	0.142	46.80	42.01	15.46
‘Sweet Repose’	0.756	0.166	0.142	46.83	50.48	15.98
‘Centenaire de Lourdes’	0.743	0.192	0.140	44.02	43.09	10.08
KORgosumo; ‘Gelber Engel’	0.726	0.178	0.137	57.17	58.61	26.06
‘Godewind’	0.803	0.166	0.134	43.64	45.66	7.12

# spectrum red: h_33_*: 0°; cold hues: h_33_* −180° → 0°; warm hues: 0° → +180°.

**Table 5:** The 25 items with the highest measured OV_hip_ values, together with the year of measurement, the corresponding Quantity and Quality Indices, and the calculated OV_hip_ values. Cultivars and taxa marked with an asterisk flower only once during the growing season.

Registration Name Followed by ARS Trade Name if the Two Differ	Year	Quantity Index	Quality Index	OV_hip_
* Rosa canina L. ‘Inermis’	2022	0.940	0.801	0.753
* Rosa micrantha Borrer ex Sm.	2017	0.936	0.801	0.750
* ‘Fragezeichen’	2017	0.888	0.801	0.711
KORorbe; ‘Apricot Vigorosa’	2017	0.911	0.485	0.442
MEIranovi; ‘Candy Rose’	2017	0.886	0.485	0.430
‘Sympathie’	2022	0.885	0.485	0.429
‘Sympathie’	2017	0.863	0.485	0.418
‘Buisman’s Glory’	2017	0.817	0.485	0.396
‘Méphisto’	2022	0.814	0.485	0.395
* Rosa micrantha Borrer ex Sm.	2018	0.936	0.364	0.341
DELamo; ‘Cathedral Bells’	2018	0.934	0.364	0.340
* Rosa micrantha Borrer ex Sm.	2022	0.909	0.364	0.331
‘Rudolph Kluis’	2017	0.880	0.364	0.321
* ‘Fragezeichen’	2018	0.873	0.364	0.318
‘Shepherd’s Delight’	2017	0.867	0.364	0.316
POULwei; ‘Nina Weibull’	2017	0.867	0.364	0.316
* ‘Fragezeichen’	2022	0.834	0.364	0.304
‘Vak Bottyán emléke’ (non-registered)	2022	0.828	0.364	0.302
DELamo; ‘Cathedral Bells’	2022	0.822	0.364	0.299
BUCbi; ‘Carefree Beauty’	2022	0.810	0.364	0.295
KORgosumo; ‘Gelber Engel’	2017	0.809	0.364	0.295
‘Cyclamen’	2017	0.807	0.364	0.294
‘Lord Stair’	2022	0.805	0.364	0.293
KORlech; ‘Lava Flow’	2018	0.804	0.364	0.293
DELstrijor; ‘Alfred Sisley’	2018	0.770	0.364	0.281

**Table 6:** Pearson correlation used to estimate the year-to-year stability of OV_hip_. In all cases, *p* < 001. The data show the correlations between years for rose hip-based ornamental value and its component indices. The weak correlation indicates that the OV_hip_ of cultivars was not stable across the years examined.

	2017	2018	2022
Quantity			
2017		0.606	0.618
2018	0.606		0.577
2022	0.618	0.577	
Quality			
2017		0.471	0.366
2018	0.471		0.389
2022	0.366	0.389	
Ornamental value			
2017		0.472	0.456
2018	0.472		0.447
2022	0.456	0.447	

**Table 7:** The 95.0 percent Tukey HSD Post Hoc test of ANOVA used to detect the effect of year. The table presents the years between which significant differences were detected for the two indices and the P_hip_ ornamental value. Years marked with an asterisk differ significantly, indicating that the differences in OV_hip_ values between those years are statistically significant rather than the result of random variation.

Quantity	Sig.	Difference	±Limits
2017–2018	*	0.026	0.015
2017–2022	*	0.032	0.014
2018–2022		0.006	0.015
Quality			
2017–2018	*	−0.071	0.034
2017–2022		−0.027	0.032
2018–2022	*	0.045	0.033
Ornamental Value			
2017–2018	*	0.012	0.012
2017–2022	*	0.019	0.011
2018–2022		0.006	0.011

**Table 8:** Average OV_hip_ of cultivar groups in the Budatétény Rose Garden, the number of measured items within each group (where greater than 1), and the item with the highest decorative value in each group.

Groups	Average OV_hip_	Number of Measured Items	Items with the Highest OV_hip_ Within Group (Registration Name)
Species	0.297	6	*Rosa micrantha* (0.75)
Lucieae (Wichuraiana) hybrids	0.223	3	‘Fragezeichen’ (0.44)
Kordesii hybrids	0.121	5	‘Sympathie’ (0.36)
Albas	0.087	2	‘Alba Maxima’ (0.15)
Miniatures	0.074	5	‘Libán’ (non-registered) (0.18)
Shrubs	0.067	32	MEIranovi (0.33)
Hulthemia hybrids	0.063	6	INTercombig (0.13)
Climbing Floribundas	0.063	4	‘Fred Loads’ (0.08)
Floribundas	0.059	206	‘Méphisto’ (0.28)
Polyanthas	0.056	19	‘Rudolph Kluis’ (0.25)
Portlands	0.051	3	‘Rose de Rescht’ (0.10)
Chinensis hybrids	0.048	2	‘Grüss an Teplitz’ (0.08)
Bourbons	0.046	3	‘Louise Odier’ (0.08)
Modern Climbings	0.046	36	‘White Dawn’ (0.22)
Climbing Hybrid Teas	0.040	7	‘Dame de Coeur, Clg.’ (0.12)
Grandifloras	0.039	23	‘June Bride’ (0.12)
Gallicas	0.031	5	*Rosa gallica* officinalis (0.08)
Hybrid Teas	0.026	319	DELamo (0.22)
Hybrid perpetuals	0.019	3	‘Urdh’ (0.02)
Damasks	0.014	5	‘Kazanlik’ (0.02)
Mosses	0.004	2	‘Crested Moss’ (0.01)

## Data Availability

The raw data supporting the conclusions of this article will be made available by the authors on request.
